# Expression of leukotriene receptors in the rat dorsal root ganglion and the effects on pain behaviors

**DOI:** 10.1186/1744-8069-6-57

**Published:** 2010-09-17

**Authors:** Masamichi Okubo, Hiroki Yamanaka, Kimiko Kobayashi, Tetsuo Fukuoka, Yi Dai, Koichi Noguchi

**Affiliations:** 1 Department of Anatomy and Neuroscience, Hyogo College of Medicine, 1-1 Mukogawa-cho, Nishinomiya, Hyogo 663-8501, Japan; 2 Department of Pharmacy, School of Pharmacy, Hyogo University of Health Sciences. Kobe, Hyogo 650-8530, Japan

## Abstract

**Background:**

Leukotrienes (LTs) belong to the large family of lipid mediators implicated in various inflammatory conditions such as asthma and rheumatoid arthritis. Four distinct types (BLT1, BLT2, CysLT1 and CysLT2) of G-protein-coupled receptors for LTs have been identified. Several studies have reported that LTs are involved in inflammatory pain, but the mechanism and the expression of LT receptors in the nociceptive pathway are unknown.

**Results:**

We investigated the precise expression of these four types of LT receptors in the adult rat dorsal root ganglion (DRG) using reverse transcription-polymerase reaction (RT-PCR) and radioisotope-labeled *in situ *hybridization histochemistry (ISHH). We detected mRNAs for BLT1 and CysLT2 in the DRG, but not for BLT2 and CysLT1. CysLT2 mRNA was preferentially expressed by small sized DRG neurons (about 36% of total neurons), whereas BLT1 mRNA was expressed by non-neuronal cells. Double labeling analysis of CysLT2 with NF-200, calcitonin gene-related peptide (CGRP), isolectin B4 (IB4), transient receptor potential vanilloid subfamily 1 (TRPV1) and P2X3 receptor revealed that many CysLT2-labeled neurons were localized with unmyelinated and non-peptidergic neurons, and interestingly, CysLT2 mRNA heavily co-localized with TRPV1 and P2X3-positive neurons. Intraplantar injection of LTC4, a CysLT2 receptor agonist, itself did not induce the thermal hyperalgesia, spontaneous pain behaviors or swelling of hind paw. However, pretreatment of LTC4 remarkably enhanced the painful behaviors produced by alpha, beta-methylene adenosine 5'-triphosphate (αβ-me-ATP), a P2X3 receptor agonist.

**Conclusions:**

These data suggests that CysLT2 expressed in DRG neurons may play a role as a modulator of P2X3, and contribute to a potentiation of the neuronal activity following peripheral inflammation.

## Background

The leukotrienes (LTs) are a family of biologically active lipid mediators. They are synthesized from arachidonic acid (AA) *via *the 5-lipoxygenase pathway. AA is enzymatically converted to LTB4, LTC4, LTD4 and LTE4 that are known as bioactive LTs. LTC4, LTD4 and LTE4 are collectively termed the cysteinyl leukotrienes (CysLTs). LTs are peripherally produced by activated leukocytes in response to peripheral inflammation, such as asthma and atopic dermatitis [1,2]. Four different types (BLT1, BLT2, CysLT1 and CysLT2) of G-protein-coupled receptor for LT have been cloned [3-6]. LTB4 activates BLT1 and BLT2, and CysLTs activate CysLT1 and CysLT2.

Peripheral inflammation often elicits mechanical and thermal hyperalgesia. The most studied of these lipid mediators are the prostaglandins (PGs) of the cyclooxygenase pathway of AA metabolism [7,8]. Expression of G-protein-coupled receptors of EP for E-type PG is localized in C-fibers, unmyelinated nociceptive fibers, in the dorsal root ganglion (DRG) [8]. Activation of EP signaling plays a role in neuronal sensitization mediating modulation of the transient receptor potential vanilloid subfamily 1 (TRPV1) receptor and P2X3 receptor [9,10].

Intradermal injection of LTB4 has been shown to produce both thermal and mechanical hyperalgesia [11,12]. Jain et al. have reported that LTs are involved in inflammatory pain induced by carrageenan [13]. Furthermore, we demonstrated that an increase in LT synthesis in microglia in the spinal cord induced by peripheral nerve injury contributes to neuropathic pain [14]. However, in the periphery, the mechanism of the nociception induced by LTs is unknown and the precise expression pattern of LT receptors in the DRG has not been clarified. The purpose of this study is to examine the expression of LT receptor mRNAs in the DRG to assess whether LT receptors are expressed in nociceptive neurons. Furthermore, we attempted to determine the nociceptive role of LT receptors in DRG by behavioral analyses.

## Results

### Expression of LT receptors in the DRG

**Figure 1:**
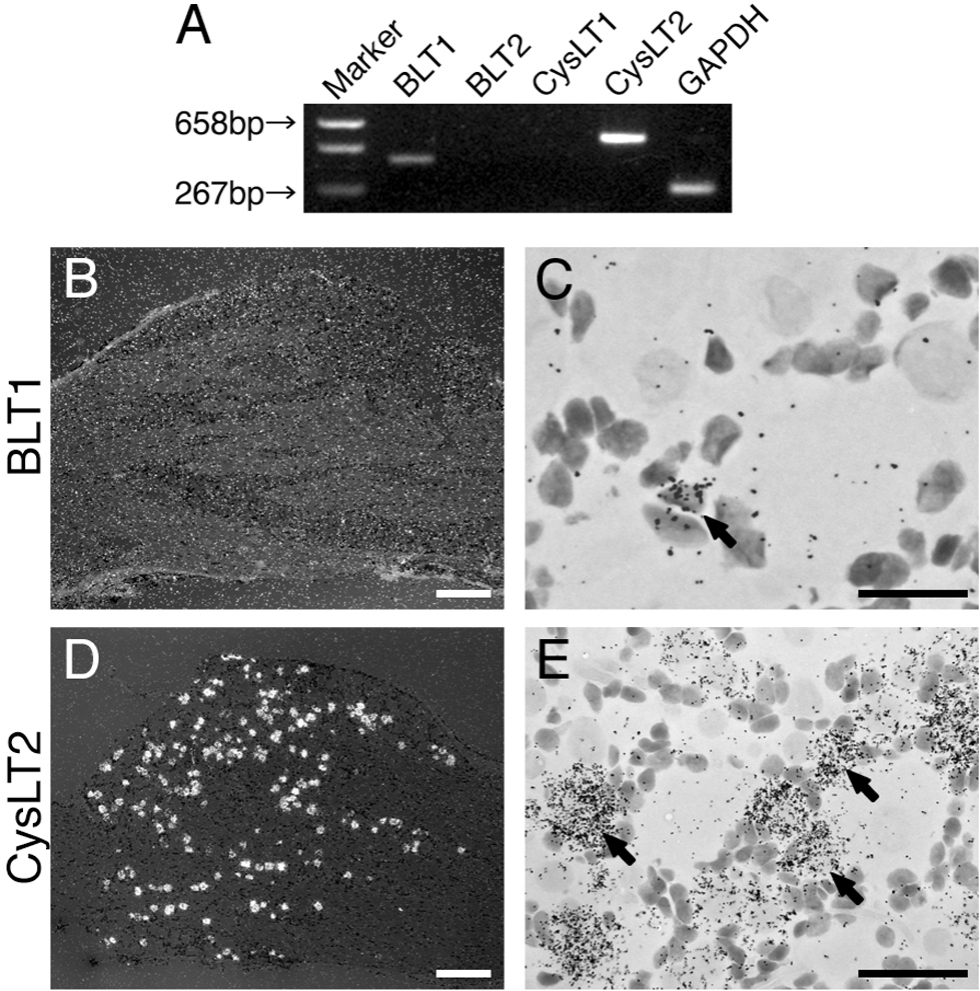
**Expression of LT receptor mRNAs in the rat DRG**. (A) The expression of mRNAs for LT receptors were determined by the RT-PCR technique. Gel panel shows PCR products from the L4, 5 DRGs taken from naive rats. (B, D) Low-magnification darkfield images of ISHH show BLT1 and CysLT2 mRNAs of naive rats, respectively. (C, E) Higher-magnification brightfield images of the left-hand images. Arrows indicate positively labeled cells by ISHH. Scale bars: B, D; 500 μm, E; 25 μm, C; 12.5 μm.

**Figure 2:**
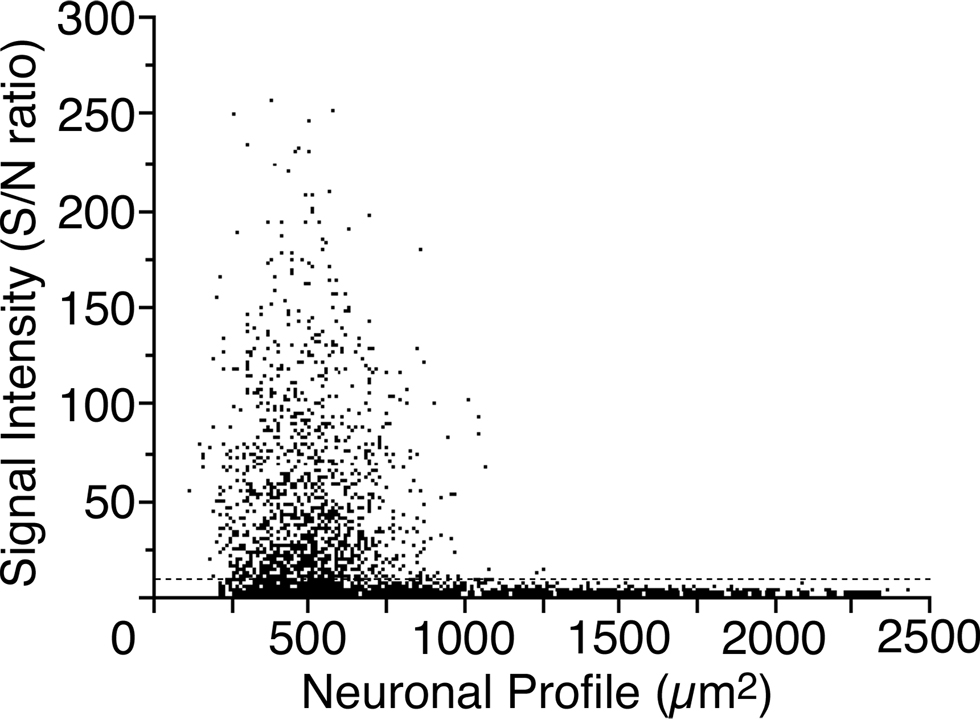
**Scatterplot diagram of the DRG neurons expressed mRNA for CysLT2**. Individual cell profiles are plotted according to the cross-sectional area and signal intensity (n = 4, 2819 cells). The dashed line indicates the borderline between the negatively and positively labeled neurons (S/N ratio = 20).

To examine whether sensory neurons express the LT receptor mRNAs, we performed reverse transcription-polymerase chain reaction (RT-PCR) and *in situ *hybridization histochemistry (ISHH) using adult rat DRG. The mRNAs for BLT1 and CysLT2 mRNAs were expressed in the DRG, but not the BLT2 and CysLT1 mRNAs (Figure 1A). For the ISHH, the BLT1 mRNA was expressed in an extremely limited population of non-neuronal cells (Figure 1B, C). With brightfield imaging of ISHH for the BLT1 mRNA, silver grains were accumulated over the non-neuronal cells whose nuclei were heavily stained with hematoxylin (Figure 1C). In contrast to the BLT1 mRNA, a subpopulation of DRG neurons expressed CysLT2 mRNA (Figure 1D, E). The darkfield photograph displayed distinguishable clusters of silver grains over the tissue with minimal background signals (Figure 1D). The brightfield and high magnification images confirmed the presence of CysLT2 on neuronal cell bodies (Figure 1E). To evaluate objectively the expression of the CysLT2 mRNA in DRG neurons, we measured, calculated, and plotted the signal-to-noise (S/N) ratio and cross-sectional area of each neuron (Figure 2). Based on this scattergram, neuronal profiles with a grain density of 20-fold the background level or higher (S/N ratio > 20) were considered positively labeled for this mRNA. With this criterion, 35.8 ± 3.3% of profiles were positively labeled for CysLT2 mRNA of the total DRG neurons (Table 1). The scattergram revealed that CysLT2 mRNA was expressed more intensely by the neurons with cell profiles less than 600 μm^2 ^compared with the medium or large-size neurons. The size distribution of the positively labeled profiles for CysLT2 mRNA is shown in Table 1. The CysLT2 mRNA was expressed in a limited population of small (< 600 μm^2^) and medium-size (600-1200 μm^2^) neurons, whereas large-size (> 1200 μm^2^) neurons were not labeled for this mRNA (Table 1). The neuronal size definition was described previously [15].

**Table 1:** Distribution of CysLT2 mRNA Expression in the DRG

	Small	Medium	Large
	(< 600 μm^2^)	(600-1200 μm^2^)	(> 1200 μm^2^)
CysLT2	76.5 ± 6.5	23.5 ± 6.5	0.0 ± 0.0

Percentages of CysLT2 mRNA-expressing cells in total positive cells.

Mean ± SEM (n = 4).

### Characterization of CysLT2-labeled neurons

**Figure 3:**
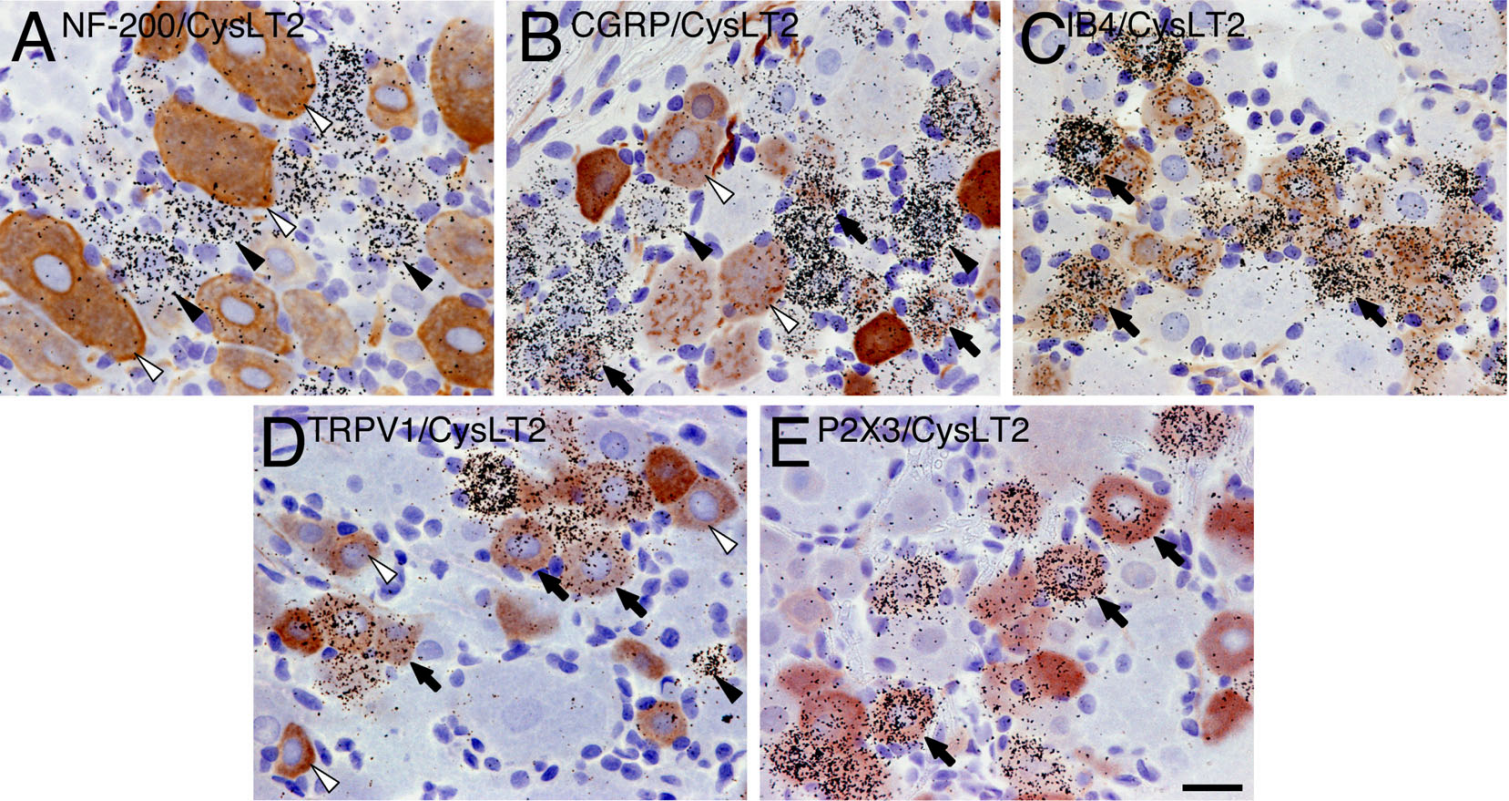
**Distribution of CysLT2 mRNA in histochemically identified neuronal subpopulations in the rat DRG**. Brightfield images of combined immunohistochemistry for (A) NF-200, (B) CGRP, (C) IB4, (D) TRPV1, (E) P2X3 with ISHH for CysLT2 mRNA. Arrows indicate examples of double-labeled cells. Solid arrowheads indicate positively labeled cells by ISHH and open arrowheads indicate examples of immunoreactive cells. Scale bars; 25 μm.

To characterize the expression of CysLT2 mRNA in DRG neurons, we used double labeling ISHH with immunohistochemistry (IHC) for NF-200, a maker of myelinated A-fiber neurons. We found NF-200-immunoreactive neurons in 36.3 ± 1.5% of the total neurons (Table 2). No specific staining was observed in the absence of the primary antibody (data not shown). The results of double labeling analysis of CysLT2 mRNA with NF-200 showed that 9.6 ± 3.4% of the CysLT2 mRNA-positive profiles expressed NF-200, conversely, 8.0 ± 2.3% of NF-200-profiles expressed CysLT2 mRNA (Figure 3A; Table 2). The CysLT2 mRNA was expressed in 44.0% of NF-200 negative profiles, which were considered unmyelinated neurons (C-fiber). We tested the co-expression of CysLT2 mRNA with CGRP and IB4 in order to identify the peptide-dependent neuronal subpopulations [16], using double labeling of ISHH with IHC. We observed CGRP-immunoreactive and IB4-binding neurons in 39.0 ± 3.1% and 37.5 ± 2.9% of the total neuronal profiles, respectively (Table 2). The results of the double labeling analysis of CysLT2 mRNA with CGRP and IB4 showed that 27.5% of the CysLT2 mRNA-positive profiles expressed CGRP; conversely, 25.6% of CGRP-profiles expressed CysLT2 mRNA (Figure 3B, Table 2), and 85.6% of the CysLT2 mRNA-positive profiles expressed IB4, conversely, 82.0% of IB4-profiles expressed CysLT2 mRNA (Figure 3C, Table 2). These results indicated that CysLT2 mRNA was expressed in non-peptidergic neurons rather than peptidergic neurons.

**Table 2:** Percentages of Colocalization of CysLT2 mRNA with NF-200, CGRP, IB4, TRPV1 and P2X3 Immunoreactive Neurons in DRG

*y*	*x/y*	*y/x*
NF-200 (36.3%)	8.0 ± 2.3	9.6 ± 3.4
CGRP (39.0%)	25.6 ± 3.0	27.5 ± 3.9
IB4 (37.5%)	82.0 ± 4.6	85.6 ± 1.5
TRPV1 (36.7%)	69.6 ± 4.6	71.2 ± 1.8
P2X3 (34.0%)	88.8 ± 2.2	80.7 ± 3.7

x/y means the percentage of CysLT2 (x) mRNA-expressing cells in y immunoreactive cells. (number); means percentage of the neurons expressing the corresponding immunoreactivity in total DRG neurons. Mean ± SEM (n = 4).

Next, to examine whether CysLT2 mRNA was co-expressed with TRPV1 and P2X3 that are considered as pivotal nociceptors in primary afferent fibers, we tested the percentage of colocalization of CysLT2 mRNA with TRPV1 and P2X3. We observed TRPV1 and P2X3-ir neurons in 36.7 ± 1.5% and 34.0 ± 1.9% of the total neuronal profiles, respectively (Table 2). Further, 71.2% of the CysLT2 mRNA-positive profiles expressed TRPV1; conversely, 69.6% TRPV1- positive profiles expressed CysLT2 mRNA (Figure 3D; Table 2) and 80.7% of the CysLT2 mRNA-positive profiles expressed P2X3; conversely, 88.8% P2X3- positive profiles expressed CysLT2 mRNA (Figure 3E; Table 3).

**Table 3:** Sequence Location of Primers Used in This Study

*Primer*			
**Gene**	**GenBank Accession no.**	**Forward**	**Reverse**

BLT1	AB025230	1812-1831	2231-2212
BLT2	AB052660	488-507	939-920
CysLT1	AB052685	234-253	698-679
CysLT2	AB052661	105-124	670-651
GAPDH	M17701	80-99	350-331

### Effect of LTC4, a CysLT2 receptor agonist, on pain-related behaviors

**Figure 4:**
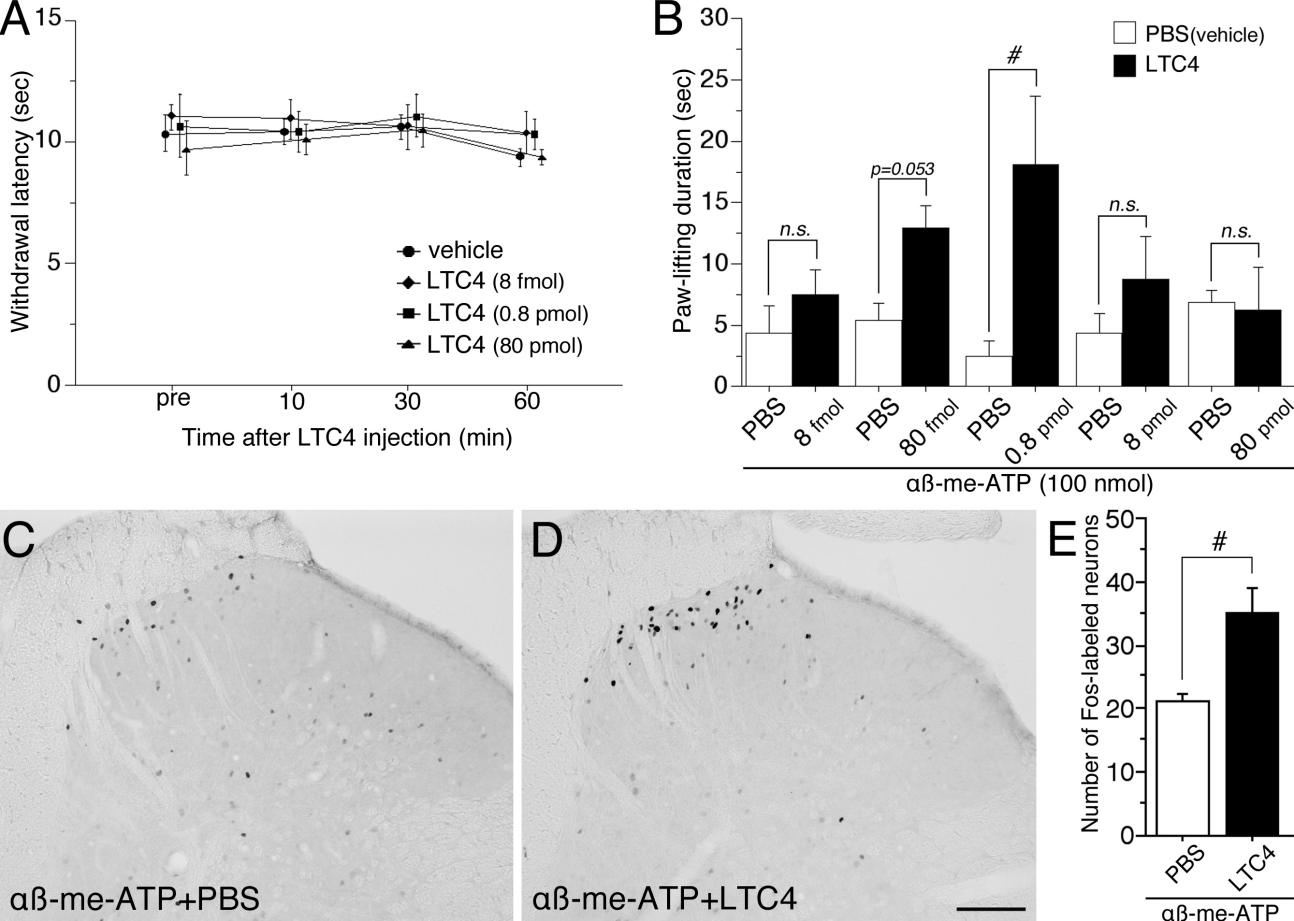
**Effect of LTC4, a CysLT2 receptor agonist, on αβ-me-ATP induced pain behaviors and Fos expression**. (A) Thermal sensitivity was evaluated by measuring withdrawal latency (sec) at pre (pretreatment), 10, 30 and 60 min after intraplantar injection of LTC4 using a plantar test. (B) The nocifensive behaviors induced by αβ-me-ATP (100 nmol) were evaluated by measuring paw-lifting duration (sec). LTC4 was given at several doses of 8 fmol - 80 pmol/paw, and the ensuing hind paw-lifting duration over the following 4 min was measured. #p < 0.05 compared with vehicle treated group. n = 4-6 for each group. (C, D) Fos protein immunoreactivity in the L5 spinal cord induced by αβ-me-ATP (100 nmol) alone (C) and LTC4 (0.8 pmol) followed by αβ-me-ATP intraplantar injection (D). (E) Number of Fos-labeled neurons in spinal laminae I-II shown in C and D. #p < 0.05 compared with vehicle treated group. n = 3 for each group. Scale bar: 100 μm.

Leukotrienes are known as proinflammatory lipid mediators, and CysLT2 was co-localized with TRPV1, a heat sensor, in DRG neurons. We examined whether LTC4, a CysLT2 receptor agonist, leads to thermal hyperalgesia (Figure 4A). We tested heat sensitivity of the hind paw after intraplantar injection of LTC4 (8 fmol, 0.8 pmol and 80 pmol). None of doses affected on heat sensitivity at 10, 30 and 60 min after LTC4 injection (Figure 4A). LTC4 alone (0.8 pmol) did not contribute to the nocifensive behaviors (pain-like behaviors) and swelling of the hind paw (data not shown).

Next, because CysLT2-positive cells heavily co-localized with P2X3, we examined whether intraplantar injection of LTC4 can enhance the nocifensive behaviors induced by alpha, beta-methylene adenosine 5'-triphosphate (αβ-me-ATP), a P2X3 receptor agonist. In normal rats, αβ-me-ATP (100 μmol) consistently induced periods of intermittent hind paw-lifting behavior, which mostly began within 30-40 s after the injection and continued for the first 4 min [17]. Intraplantar injection of LTC4 at 0.8 pmol before the αβ-me-ATP injection induced a remarkable increase of paw-lifting behaviors (Figure 4B). The increase of duration of paw lifting was significantly larger than that after the injection of PBS plus αβ-me-ATP (Figure 4B). Lower and higher doses of LTC4 (< 80 fmol and 8 pmol <) did not show the alteration of nocifensive behaviors by αβ-me-ATP injection (Figure 4B). Potentiation of nocifensive behaviors induced by LTC4 showed a bell-shaped concentration-effect curve, with no significant effect at lower and higher amounts.

### Pretreatment with the LTC4 increased αβ-me-ATP-induced Fos expression

A single injection of αβ-me-ATP (100 nmol) induced Fos expression in a small number of spinal neurons (Figure 4C). The labeled neurons were in the superficial dorsal horn but were relatively distributed throughout the spinal cord laminae. The injection of αβ-me-ATP (100 nmol) into the hind paw of the LTC4 (0.8 pmol)-pretreated rats induced elevated Fos expression in spinal neurons (Figure 4D). The Fos-labeled cells were prominently observed in the medial half of the superficial laminae of the spinal dorsal horn. The number of Fos-labeled cells in laminae I-II induced by the injection of αβ-me-ATP (100 nmol) in rats pretreated LTC4 (0.8 pmol) was significantly larger (almost 1.5 times) than those pretreated with PBS (Figure 4E).

## Discussion

LTs are lipid mediators with a proinflammatory profile and have been implicated in the pathogenesis of several types of inflammation [1]. For example, the blood and synovial fluids of patients with rheumatoid arthritis contain higher levels of LTB4 than people without rheumatoid arthritis [18]. LTB4 is known as a potent neutrophil chemotactic agent. It is considered that the neutrophils that are infiltrated by rheumatoid arthritis produce LTB4 in synovial fluids and induce the inflammatory condition. Several studies have demonstrated that LTs are involved in inflammatory pain [11-13]. It is well known that nerve growth factor (NGF) is up-regulated in inflammatory tissue and sensitizes nociceptors [19] leading to thermal hyperalgesia [20]. It has reported that NGF increased LTB4 in the rat paw skin and these results suggested the participation of LTB4 in NGF-induced local thermal hyperalgesia [21]. Furthermore, Trang et al. have reported that intrathecal administration of LTB4 leads to thermal hyperalgesia, and a BLT1 receptor antagonist suppresses this hyperalgesia [22]. These previous reports indicate that LTs in peripheral tissues may have an effect on primary afferents.

In the present study, we demonstrated the expression of LT receptors, BLT1, BLT2, CysLT1, and CysLT2, in the adult rat DRG. We could not detect BLT2 and CysLT1 mRNAs in the DRG. We found the BLT1 mRNA expression in non-neuronal cells, but Andoh et al. reported expression of BLT1 in mouse DRG neurons [23]. This discrepancy may be due to the difference of the species (rat versus mouse) or the methods (ISHH versus IHC). In contrast to the expression of BLT1 mRNA, CysLT2 mRNA was expressed in DRG neurons. CysLT2 was cloned in 2000 [5], however, there has been limited information of its tissue distribution in nervous system, such as in the astrocyte in brain [24]. CysLT2 is involved in apoptosis induced by oxygen-glucose deprivation *in vitro *[24], but its functional role remains largely unknown. We precisely quantified CysLT2 mRNA in the adult rat DRG showing that about 40% of DRG neurons expressed CysLT2 mRNA (S/N > 20) and small sized DRG neurons preferentially expressed CysLT2. Double-labeling analysis with NF-200 and CysLT2 showed that most CysLT2-labeled cells did not express NF-200. Moreover, a lot of CysLT2-positive profiles exclusively co-localized with IB4-binding, a quarter of CGRP-positive neurons expressed CysLT2 mRNA. These results indicate that CysLT2 was mainly expressed in unmyelinated and non-peptidergic neurons.

Interestingly, CysLT2 mRNA expressing neurons were heavily co-localized with TRPV1- or P2X3-positive neurons. TRPV1, one of the TRPV family, has been cloned and is a thermosensitive channel with a threshold of 42 degrees Celsius [25]. TRPV1 is expressed in small sized neurons [26] and is modulated by various G-protein coupled receptors, such as EP4 [8], protease-activated receptor 2 [27] and neurokinin-1 receptor [28]*via *the protein kinase C (PKC) pathway. 12-(S)-HPETE, a product of 12-lipoxygenase, potentiates the TRPV1 current in HEK cells [29]. Thompson et al. have reported that the signaling pathway for CysLT2 is involved in the activation of PKC pathway *via *Gq-proteins [30]. Because it is possible that CysLT2 can sensitize TRPV1 in primary sensory neurons, we examined whether intraplantar injection of LTC4 leads to thermal hyperalgesia. All doses of LTC4 (8 fmol, 0.8 pmol and 80 pmol) did not affect on heat sensitivity at 10, 30 and 60 min after the injection in normal rats. The data indicate LTC4 does not have a role on thermal hyperalgesia in a normal condition. However, a further study is required to know the role of LTC4 on thermal sensitivity in tissue inflammation.

P2X3 is a ligand-gated ion channel for ATP, and belongs to P2X family. P2X3 is of particular interest in the context of pain pathways, because it is selectively expressed at high levels by nociceptors [31], and electrophysiological studies suggest that the P2X receptors in sensory neurons may play an important role in the generation and/or modulation of the pain signaling from the periphery to the spinal cord [32]. Furthermore, we previously reported that P2X3 in peripheral afferents plays a role in the induction of the hypersensitivity to mechanical stimulation observed during peripheral inflammation [33] and many P2X3s are co-expressed with protease-activated receptor 2 in the rat dorsal root ganglion neurons. Nocifensive behaviors induced by αβ-me-ATP injection to the hind paw were significantly augmented after the application of protease-activated receptor 2 agonists [17]. Fos expression induced by the αβ-me-ATP injection in dorsal horn neurons was also increased after the pre-application of protease-activated receptor 2 agonists [34]. These previous studies led us to behavioral experiments to study whether the LTC4 have a role in potentiation of pain sensation induced by αβ-me-ATP. Intraplantar injection of LTC4 before the αβ-me-ATP injection induced a significant increase of paw-lifting behaviors and Fos expression in the spinal dorsal horn. Based on the finding described in the present study, we concluded that CysLT2, the receptor of LTC4, located in the primary afferent, might modulate the activation of P2X3 by the injection of αβ-me-ATP.

## Conclusions

We found that the CysLT2 is preferentially expressed by small-sized, non-peptidergic and nociceptive neurons expressing TRPV1 or P2X3 in the DRG, and contribute to the potentiation of pain behaviors induced by αβ-me-ATP. Our current observations in the context of previous findings may indicate a novel functional role of CysLT2 in the peripheral nervous system.

## Methods

### Experimental animals

Male Sprague-Dawley rats weighing 200-250 g were used as subjects. All animal experimental procedures were approved by the Hyogo College of Medicine Committee on Animal Research and were performed in accordance with the National Institutes of Health guidelines on animal care. Rats were used for the behavioral analyses. A few minutes after unilateral intraplantar injection of leukotriene C4 (LTC4, Cayman chemical, Ann Arbor, MI) [5S-hydroxy-6R-(S-glutathionyl)-7E,9E,11Z,14Z-eicosatetraenoic acid] [0.8 pmol-8 nmol in 50 μl of phosphate-buffered saline (PBS)], an agonist of CysLT2 receptor, the rats received intradermal injection of αβ-me-ATP (100 nmol, Sigma, St Louis, Missouri, USA) in 50 μl PBS to the plantar surface of the left hind paw. The rats were placed in a wire mesh cage immediately after the injection, and the duration of hind paw lifting during the first 4 min were measured [17,35]. For measurement of thermal hyperalgesia, the withdrawal latency (sec) of hind paw was measured from 10 to 60 min after intraplantar injection of LTC4. Thermal hyperalgesia was assessed with a plantar test (7370, Ugo Basile, Comerio, Italy). The detailed method of thermal sensitivity measurement in rat hind paw was described previously [36].

### Reverse transcription-polymerase chain reaction (RT-PCR) and *in situ *hybridization histochemistry (ISHH)

The rats were killed by decapitation under deep ether anesthesia. L4 and L5 DRGs were removed and rapidly frozen with powdered dry ice and stored at 80°C until use. Extraction of total RNA was done by the single step extraction method using ISOGEN (Nippon Gene, Tokyo, Japan) that was described in a previous paper [37]. The forward and reverse primers specific for rat BLT1, BLT2, CysLT1, CysLT2 and GAPDH were designed as shown in Table 3. Amplification cycle were 33 for each cDNA. The amplified cDNA was cloned into p-GEM T-easy (Promega, MI, USA) and sequenced. These clones were used to generate the cRNA probes for ISHH.

For ISHH, the rats were killed by decapitation under deep ether anesthesia. The bilateral L4 and L5 DRGs were dissected out, rapidly frozen in powdered dry ice, and cut on a cryostat at 5 μm thickness. The protocol for ISHH was base on a publish method [38]. Using the enzyme-digested cloned, α^35^S UTP-labeled antisense and sense cRNA probe were synthesized. Theα^35^S-labeled probes in hybridization buffer were placed on the section, and then incubated at 55°C overnight. Sections were then washed and treated with 1 μg/ml RNase A. Subsequently, sections were dehydrated and air-dried. After the hybridization reaction, the slides were coated with NTB emulsion (Kodak, Rochester, NY, USA) and exposed for 3-4 weeks. Once developed in D-19 (Kodak), the sections were stained with hematoxylin-eosin and coverslipped.

### Double labeling analysis of ISHH with immunohistochemistry (IHC)

For double labeling of ISHH with IHC, the rats were deeply anesthetized with sodium pentobarbital (70-80 mg ⁄ kg body weight, i.p.) and perfused transcardially with 100 ml of 1% paraformaldehyde in 0.1 M phosphate buffer, pH 7.4, followed by 500 ml of 4% paraformaldehyde in 0.1 M phosphate buffer. The L4 and L5 DRGs were dissected out and post-fixed in the same fixative for 4 h at 4°C, followed by immersion in 30% sucrose in 0.1 M phosphate buffer at 4°C overnight. The tissue was frozen in powdered dry ice and cut on a cryostat at 5 μm thickness. The sections were processed for IHC using the ABC method [39]. Following antibodies and binding protein were used: Mouse anti-NF200 monoclonal antiserum (1:40000, Sigma, St. Louis, MO, USA), rabbit anti-CGRP (1:10000, Amersham, Buckinghamshire, UK), isolectin B4 from *Griffonia simplicifolia *(IB4, 1:200, Sigma, St. Louis, MO, USA), rabbit anti-TRPV1 (1:100, Oncogene, Cambridge, MA, USA) and rabbit ant-P2X3 (1:500, Oncogene, Cambridge, MA, USA). The sections were washed in TBS and then incubated in biotinylated anti-rabbit or anti-mouse IgG (1:400; Vector Laboratories, Burlingame, CA, USA) in Tris buffer saline (TBS; Tris-HCl 0.1 M, NaCl 0.15 M) containing 5% serum for 2 h at 4°C, followed by incubation in avidin-biotin-peroxidase complex (Elite ABC kit; Vector, CA, USA) for 1 h at room temperature. The horseradish peroxidase reaction was developed in TBS, pH 7.4, containing 0.05% 3,39-diaminobenzidine tetrahydrochloride (Wako, Tokyo, Japan) and 0.01% hydrogen peroxidase. Sections were then washed in TBS and used for ISHH.

### Immunohistochemistry for Fos expression

For Fos protein immunohistochemistry, rats were divided into two experimental groups; group 1: rats received injection of αβ-me-ATP and PBS, and were perfused 2 h after the injection; group 2: rats received injection of αβ-me-ATP and LTC4 (80 pmol) and were perfused 2 h after the injection. After appropriate survival times, the rats were deeply anesthetized and perfused transcardially with 4% paraformaldehyde described in double labeling method. L4/L5 segments of the spinal cord were removed for immunohistochemistry as described previously [40]. Rabbit primary antibody for Fos (1:20000; Ab-5; Oncogene) was used. The number of Fos-labeled neurons in laminas I-II was counted in randomly selected sections (ten out of 18-28 sections per rat). A labeled nucleus was judged as positively labeled only when a structure of appropriate size and shape indicated a clear increase in immunoreactivity above the background, but without considering intensity of the staining.

### Quantitative analysis

Measurements of the density of silver grains over randomly selected tissue profiles were performed using a computerized image analysis system (NIH Image, version 1.61), where only neuronal profiles that contained nuclei were used for quantification. At a magnification of 200× and with bright-field illumination, upper and lower thresholds of gray level density were set such that only silver grains were accurately discriminated from the background in the outlined cell or tissue profile and read by the computer pixel-by-pixel. Subsequently, the area of discriminated pixels was measured and divided by the area of the outlined profile, giving a grain density for each cell or tissue profile. To reduce the risk of biased sampling of the data because of varying emulsion thickness, we used a signal-to-noise (S/N) ratio for each cell in each tissue. The S/N ratio of an individual neuron and its cross-sectioned area, which was computed from the outlined profile, was plotted. Based on this scatter gram, neurons with a grain density of ten-fold the background level or higher (20 < S/N ratio) were considered positively labeled for CysLT2 mRNA. Because a stereological approach was not used in this study, quantification of the data may represent a biased estimate of the actual numbers of neurons. At least 500 neurons from the L4/5 DRG of each rat were measured. The number of positively labeled DRG neurons was divided by the number of neuronal profiles counted in each DRG. For IHC, only the signals that were clearly discriminative immunoreactive profiles were considered as the positive expressions.

## Competing interests

The authors declare that they have no competing interests.

## Authors' contributions

MO with KK and HY designed and performed all of experiments, analyzed data and drafted the paper. HY, KK, TF, YD and KN supervised the project and edited the manuscript. All authors contributed to data interpretation, have read and approved the final manuscript.
